# Near‐Field Acoustic Imaging Using Fiber‐Optic Distributed Acoustic Sensing and Beamforming Techniques

**DOI:** 10.1002/advs.202512513

**Published:** 2025-11-14

**Authors:** Marcelo A. Soto, Diego Badillo‐San‐Juan

**Affiliations:** ^1^ Department of Electronics Engineering Universidad Técnica Federico Santa María Valparaíso 2390123 Chile

**Keywords:** acoustic imaging, beamforming, distributed acoustic sensing, optical fiber sensors, rayleigh scattering

## Abstract

Distributed acoustic sensors (DAS) detect mechanical vibrations along optical fibers with meter‐scale spatial resolution, capturing the waves directly reaching the fiber. Deploying dense fiber nets in two or three dimensions for spatially‐precise sensing over large regions is often impractical or unfeasible, highlighting the need for a method to monitor the propagating mechanical (acoustic) waves in regions far from the sensing fiber. Here, a beamforming‐based method is proposed to image acoustic emissions around an optical fiber, including areas without fiber deployment. By combining DAS measurements from multiple sampled fiber locations (DAS channels) and using a near‐field array signal processing approach, the technique can enable the development of an acoustic camera to generate 2D or 3D acoustic maps with meter‐scale spatial resolution, covering areas spanning several square kilometers, even outside the optical fiber deployment region. The method includes a dedicated blind selection of high‐quality DAS channels to address intrinsic DAS limitations (e.g., uneven longitudinal response and directional axial response) and external factors (e.g., poor fiber‐ground coupling and multipath wave propagation). The approach allows the precise localization of vibration sources, and potentially of acoustic reflections, extending DAS capabilities to analyze the propagation of vibration waves in regions distant from the fiber.

## Graphical abstract

This work introduces a beamforming‐based method to generate acoustic images from fiber‐optic distributed acoustic sensing (DAS) data. By performing 2D scanning around the sensing fiber, the approach enables near‐field imaging and localization of acoustic sources, even in regions distant from the fiber. A data‐driven DAS channel selection improves image quality and robustness, addressing intrinsic DAS limitations and real‐world propagation challenges.

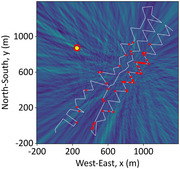

## 1 Introduction

Distributed optical fiber sensors^[^ ^1^ ^]^ have gained significant attention over the years for their ability to provide spatially resolved monitoring of environmental variables such as temperature, strain, deformation, pressure, and others. These sensors use an optical fiber as both transmission line and sensing element, with environmental changes modulating light propagation properties. Among various technologies,^[^ ^1^, ^2^, ^3^, ^4^, ^5^, ^6^, ^7^, ^8^, ^9^ ^]^ distributed acoustic sensors (DAS)^[^ ^10^, ^11^ ^]^ have gained prominence in the last decade due to their capability to measure the amplitude, frequency, and phase of mechanical (acoustic) vibrations, enabling a wide range of applications. Distributed acoustic sensors^[^ ^10^, ^11^ ^]^ exploit the optical interference from the coherent Rayleigh backscattering^[^ ^12^, ^13^ ^]^ along the sensing fiber. External vibrations change the refractive index of the fiber, leading to phase shifts in the Rayleigh backscattered light. These phase shifts can be detected using phase‐sensitive interrogation methods employing coherent light,^[^ ^12^, ^13^ ^]^ in either the time^[^ ^14^ ^]^ or frequency^[^ ^15^ ^]^ domain, to quantify the induced strain.

DAS systems offer rapid response, wide acoustic bandwidth, and the ability to monitor thousands of spatial points along extended optical fibers, making them well‐suited for applications such as early landslide detection,^[^ ^16^ ^]^ urban noise monitoring,^[^ ^17^ ^]^ and seismic wave recording,^[^ ^18^ ^]^ even in deep‐ocean environments.^[^ ^19^ ^]^ DAS has also proven useful in detecting microseismic events during hydraulic fracturing operations^[^ ^20^ ^]^ and in geothermal fields.^[^ ^21^, ^22^ ^]^ However, DAS can only detect acoustic waves that actually reach the optical fiber itself. Therefore, monitoring large areas requires the fiber to be physically deployed across the region of interest, which is often impractical. Moreover, trade‐offs between spatial resolution, acoustic bandwidth, and sensitivity (strain resolution) limit the maximum length of the sensing fiber and thus the area or volume that can be covered.

Array signal processing^[^ ^23^, ^24^, ^25^ ^]^ offers a way to overcome these limitations. In general, source localization methods fall into two categories: i) time‐difference‐of‐arrival (TDOA) methods, typically restricted to single‐source detection, and ii) array signal processing techniques, which can inherently localize multiple simultaneous sources. Beamforming, a core method in array processing, exploits the spatio‐temporal information measured by sensor arrays distributed in space to perform signal enhancement, source localization, noise reduction, dereverberation, or source separation. In this context, DAS effectively transforms an optical fiber into a large‐scale, evenly sampled, and synchronously measured microphone array.^[^ ^26^, ^27^ ^]^ Array signal processing for DAS was first demonstrated by Ku et al.^[^ ^28^ ^]^ for the localization of human footsteps 10 m away from the sensing fiber. In DAS‐based seismological applications, several studies^[^ ^17^, ^29^, ^30^, ^31^, ^32^ ^]^ have employed beamforming techniques assuming planar mechanical wave propagation. These far‐field, narrowband methods are primarily limited to estimating the direction of arrival of seismic waves and cannot provide the actual position of the acoustic source. Consequently, they can enable acoustic imaging only in the far field,^[^ ^31^, ^33^ ^]^ typically represented in polar coordinates, where the angle indicates the direction of arrival and the radius corresponds to the wave propagation velocity. Other narrowband methods like multiple signal classification (MUSIC)^[^ ^34^, ^35^ ^]^ have allowed localization with specially arranged fibers, while Doppler‐based methods using optical frequency‐domain reflectometry (OFDR) have tracked moving single‐frequency sources.^[^ ^36^ ^]^ Although effective DAS detection requires some degree of fiber deployment with angular diversity to deal with the directional (axial) response of the fiber to strain, most beamforming‐based approaches demand even more specific fiber deployment configurations.^[^ ^26^, ^31^ ^]^ Recently, near‐field array signal processing has been explored to improve DAS signal quality and localize acoustic sources using either triangulation^[^ ^26^ ^]^ or a near‐field MUSIC beamforming.^[^ ^37^ ^]^ The former is a TDOA‐based approach suitable only for single‐source localization,^[^ ^26^ ^]^ while the latter uses a short, straight optical fiber, providing a 1D array^[^ ^37^ ^]^ that is prone to symmetry ambiguities and mirroring artefacts inherently to uniform linear arrays.^[^ ^24^, ^38^ ^]^ In addition, it is important to emphasize that most beamforming methods reported in the literature, both near‐ and far‐field, have been directly applied to DAS without accounting for real‐world DAS challenges, such as the non‐uniform longitudinal DAS sensitivity, the directional (axial) strain response of DAS, and multiple acoustic wave reflections in the propagation medium.

In this work, we propose a beamforming‐based acoustic imaging method that can reconstruct the acoustic energy around optical fibers using distributed acoustic sensing measurements, even in regions without direct fiber coverage. The approach performs array‐agnostic near‐field imaging by scanning the 2D region of interest and computing time delays purely from geometric relations between each image pixel and the sensing fiber. This approach, based on array signal processing, inherently has the potential to detect and identify multiple simultaneous acoustic sources from the generated acoustic image. By using a 2D array (corresponding to an arbitrary, non‐linear fiber deployment), the method allows imaging of the entire 360° space around the optical fiber, offering a significant advantage over 1D (linear) arrays, which are limited to analyzing acoustic signals from only 180° of space.^[^ ^24^, ^38^ ^]^ This geometry‐based formulation enables accurate acoustic energy imaging with 2D fiber deployments, while also avoiding the symmetry ambiguity and mirroring artifacts that typically affect uniform linear arrays^[^ ^24^, ^38^ ^]^ (often used in previous DAS studies^[^ ^27^, ^28^, ^29^, ^30^, ^33^, ^35^, ^37^ ^]^ with straight or quasi‐straight fiber deployments). By combining DAS measurements from multiple fiber locations (DAS channels) and assuming spherical wave propagation, the method reconstructs 2D (and eventually 3D) acoustic energy maps in Cartesian coordinates. These maps are displayed as color‐coded images, similar to thermal maps, effectively acting like an acoustic camera. Unlike traditional beamforming approaches,^[^ ^23^, ^24^, ^25^ ^]^ which assume uniform sensor responses, our method explicitly addresses the uneven sensitivity of DAS systems caused by: i) the nonuniform strain coupling between the optical fiber and propagation medium, ii) the directional response of DAS to axial strain, which varies with the fiber orientation, and iii) the Rayleigh intensity fading, which, for some systems, can create blind spots in the fiber, leading to unreliable acoustic measurements. To handle these effects, we use a blind data‐driven DAS channel selection procedure that automatically identifies and excludes degraded DAS channels. This improves robustness against noise, multipath distortion, and variations in DAS directional sensitivity. This represents the first demonstration of true near‐field DAS acoustic imaging using beamforming with a dedicated DAS channel selection process to enhance measurement quality. The method has been validated under broadband excitations and real‐world mechanical propagation conditions. Experimental results show source localization errors as low as 0.37% (e.g., 2 m error at 537.4 m distance), although local velocity variations and reduced sensitivity in some fiber sections can affect accuracy, as confirmed by statistical analysis. Although this is a proof‐of‐concept using basic beamforming, the proposed framework establishes a foundation for more advanced array‐processing developments, paving the way for DAS‐based acoustic imaging in a variety of fields, including geophysics, seismology, structural health monitoring, oil and gas, materials science, border security, underwater exploration, and environmental monitoring.

## 2 Results

### 2.1 Experimental Conditions

**Figure 1:**
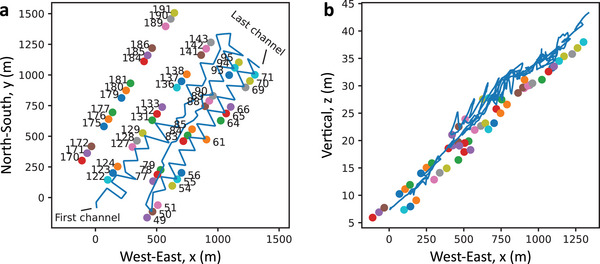
Deployment of the sensing optical fiber and acoustic source locations. The sensing optical fiber (blue line) and the 55 positions of the vibroseis track analyzed in this work (colored circles). The sensing fiber covers an area of ≈500 × 1500 m, with a vertical extent of ≈36 m, and is buried in a trench of 0.5 m depth. The first and last DAS channels (e.g., Ch. 1 and 863) are labelled for visual reference. a) Top view of the monitored area in the (*x*, *y*) plane. b) Side view of the area in the (*x*, *z*) plane.

This study uses DAS measurements from a seismic survey conducted as part of the Poroelastic Tomography (PoroTomo) project^[^ ^39^, ^40^ ^]^ at a geothermal site near Brady Hot Springs, Nevada, USA. The sensing fiber, 8.63 km long, is buried 0.5 m deep and deployed in a zigzag pattern over a 1500 m × 500 m area, as shown by the blue line in **Figure** **1**a. While mostly horizontal, the fiber exhibits vertical variations up to 36 m, as shown in Figure 1b. A commercial DAS system interrogates the fiber with a 1 kHz sampling rate, 10 m gauge length, and 1 m spatial interval, yielding 8630 acoustic channels. To ensure longitudinal independence, only channels spaced by 10 m (equal to the gauge length) are used, reducing the dataset to 863 independent DAS channels.

Seismic waves are generated by a vibroseis truck emitting 5–80 Hz chirped signals over 20 s from 200 distinct ground locations. These signals, which include both direct and reflected paths, are recorded along the fiber. The truck produces both P‐waves (longitudinal) and S‐waves (transverse), but only P‐wave data is used in this study. For statistical analysis, a subset of 55 randomly distributed source positions is selected, with their locations relative to the fiber shown and numbered in Figure 1a.

The fiber layout in Figure 1a provides a 2D array with excellent angular diversity, enabling the DAS system to detect acoustic waves from any direction within the monitored area. This helps overcome the directional sensitivity limitations of DAS, which can only measure strain along the fiber axis. Note that such a fiber deployment is commonly used to ensure reliable DAS measurements by guaranteeing that acoustic waves from all possible directions can be detected at least at some fiber locations. Although not unique to this study, the layout also breaks symmetry in wave propagation delay calculations for beamforming, avoiding the symmetry ambiguities and mirroring artifacts typical of straight‐line arrays.^[^ ^24^, ^38^ ^]^ For accurate 2D imaging, the fiber must lie within a 2D plane to resolve directional ambiguities. However, for 3D imaging, even a 2D layout is insufficient, as it cannot distinguish between waves arriving from opposite sides of the plane. Resolving these ambiguities in 3D requires sufficient fiber extension (aperture) and spatial diversity (coverage) in all three dimensions. Note that the insufficient vertical extension of the optical fiber in this case results in poor vertical spatial resolution, restricting this proof‐of‐concept demonstration to 2D imaging.

### 2.2 Data Preprocessing and Selection of DAS Channels

The proposed beamforming‐based imaging technique uses delay‐and‐sum beamforming^[^ ^24^, ^41^, ^42^ ^]^ with fiber‐optic DAS data to map the acoustic energy in the near‐field region of the DAS array. Unlike far‐field methods that assume planar wavefronts and estimate direction of arrival, this approach assumes spherical wave propagation, allowing it to focus on specific locations within a defined 2D or 3D spatial grid, essentially scanning and listening to any point in the area. Details are provided in the Experimental Section. Unlike active imaging methods (e.g., sonar or radar), which emit signals and capture reflections, this is a passive imaging method that relies solely on DAS measurements of naturally or externally emitted acoustic signals. It visualizes the acoustic power distribution in space, enabling source localization even in areas far from the sensing optical fiber.

However, directly applying delay‐and‐sum to DAS data is often ineffective^[^ ^26^ ^]^ because classical beamforming assumes uniform sensor response, a condition that is not fulfilled by DAS systems.^[^ ^17^, ^26^ ^]^ Indeed, variations in mechanical coupling, Rayleigh fading, and directional axial strain sensitivity result in nonuniform DAS channel responses. In addition, including poor‐quality channels, i.e., especially those dominated by noise or distortions from multipath reflections, can significantly degrade imaging performance. These reflections, common in anisotropic and inhomogeneous media, can increase the measured signal strength in some DAS channels signals while distorting waveforms, making the signal‐to‐noise ratio (SNR) unreliable for selecting good‐quality DAS channels.

To address this, preprocessing raw DAS data is essential. This includes digital bandpass filtering, amplitude normalization, and dynamic DAS channel selection, all performed blindly, i.e., without prior knowledge of sources or waveforms. Given that both DAS response and signal quality vary with the fiber orientation and wave arrival angle,^[^ ^17^, ^26^ ^]^ the preprocessing must be adapted dynamically for each source position in Figure 1a. After bandpass filtering (see Experimental Section), an amplitude normalization is performed to minimize the impact of the uneven DAS response on the performance of delay‐and‐sum beamforming. Unlike static calibration in microphone arrays,^[^ ^43^ ^]^ this normalization is adaptive and achieved by dividing each DAS waveform by its root‐mean‐square (RMS) value, compensating for direction‐dependent response variations and allowing the method to remain robust across varying conditions.

**Figure 2:**
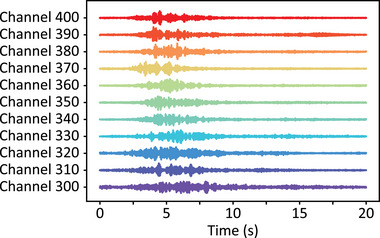
Some filtered and normalized DAS measurements. Time‐domain waveforms from some DAS channels between 300 and 400 for the vibroseis track position T180, shown after time alignment, low‐pass filtering, and normalization by their respective RMS values. The figure reveals that mostly the first seconds of the chirped mechanical wave are measured, containing mainly the low‐frequency components of the chirp. The higher frequencies of the chirped wave appear to have been attenuated during the acoustic wave propagation.

**Figure** **2** shows several DAS channels after filtering and amplitude normalization for source T180 (see Figure 1a for location). The channels primarily capture the low‐frequency components of the chirped mechanical wave, likely due to high‐frequency attenuation during propagation^[^ ^44^ ^]^ (see also Supporting Information 1). The figure verifies the different SNR levels of the DAS channels, even after the filtering and normalization, highlighting the need for channel quality evaluation before applying delay‐and‐sum beamforming.

**Figure 3:**
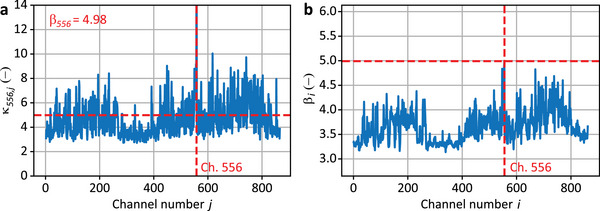
Blind evaluation of DAS channels quality for source T180. The quality of DAS channels is assessed by first calculating the phase cross‐correlation function *PCCF_ij_* between all pairs of channels (*i*, *j*) in the DAS array of *M* channels and evaluating the sharpness of the correlation peak using the local similarity indicator κ_*ij*_, as defined in the Experimental Section. This process generates an *M* × *M* matrix of indicators κ_*ij*_, where each element relates to the phase cross‐correlation between a pair of channels. Then, the global reliability indicator β_*i*_ for each channel *i* is determined as the RMS value of the 1 × *M* vector **κ**_*i*_ =  [κ_*i*,1_,  κ_*i*,2_,..,  κ_*i*,*M*_], which contains all the local reliability indicators for the channel *i* relative to the other channels. The RMS value of the vector **κ**_*i*_ is calculated over all other *j* channels, with j≠i, i.e., excluding the auto‐correlation term. The global reliability score β_*i*_ quantifies the quality of each DAS channel, with the highest β_*i*_ identifying the channel with the best quality. DAS channels are then ranked in descending order of β_*i*_ scores, allowing the selection of the top *m* channels with the highest quality. a) Values of the local similarity indicator κ_*ij*_ for the DAS channel 556, and b) global reliability score β_*i*_ for all channels, indicating that channel 556 has the highest quality for the acoustic source position T180.

The proposed channel selection method, detailed in Experimental Section, identifies high‐quality DAS channels blindly, without prior knowledge of source properties. For each DAS channel *i*, a local similarity indicator κ_*ij*_ is computed based on the phase cross‐correlation function (PCCF)^[^ ^45^ ^]^ with all other channels *j*. Note that κ_*ij*_ depends on the PCCF sharpness, making it an amplitude‐unbiased method for measuring the similarity and phase coherence between waveforms.^[^ ^45^, ^46^, ^47^ ^]^ Noisy or low‐sensitivity channels exhibit low correlation with others, while high‐quality channels (i.e., undistorted and with high SNR) show strong average correlation. To quantify the overall quality of a given channel *i*, the RMS of all κ_*ij*_ values is calculated, yielding a global score β_*i*_ (see Experimental Section). Channels with the highest β_*i*_ values, corresponding to the ones with the best quality, are selected for imaging. While similar local and global indicators have been used in a prior work,^[^ ^26^ ^]^ this approach ranks channels based on the global score β_*i*_, and not on the local indicator κ_*ij*_, as previously used for triangulation‐based source localization.^[^ ^26^ ^]^ As an example, **Figure** **3**a shows κ_*ij*_ for the DAS channel *i* = 556 for the source position T180, with β_556_  =  4.98 marked by a horizontal red line; the vertical red line indicates the analyzed DAS channel. Figure 3b displays β_*i*_ for all 863 channels, with the highest scores being selected for the acoustic imaging process (see Experimental Section for details).

### 2.3 Near‐Field Acoustic Imaging Results

To generate near‐field acoustic images from DAS measurements and localize sources around the sensing fiber, a 2D imaging area of 1600 × 1600 m is defined in the (*x*,  *y*) plane. The steered response power (see Experimental Section) is computed over a 20 s window at each scanned (*x*,  *y*) point on a 10 m resolution grid. After identifying the position with the maximum steered power, indicating a potential source location, a finer 40 × 40 m search is performed around it using a resolution cell of 1 × 1 m.

In principle, with a known average acoustic propagation velocity, delay‐and‐sum beamforming could apply time delays to the normalized DAS channels to scan the grid. Unlike classical beamforming, which steers the beam toward a single source, this method performs a full 2D scan of the imaged space. Note, however, that while the local acoustic velocity range in the area is known,^[^ ^39^, ^40^ ^]^ the exact average propagation speed varies between experiments and source locations. To address this, images are computed for 40 candidate velocities between 320 and 359 m s^−1^, covering the expected range.^[^ ^39^, ^40^ ^]^ The maximum steered power across all images determines both the estimated source location and the most likely propagation velocity.

**Figure 4:**
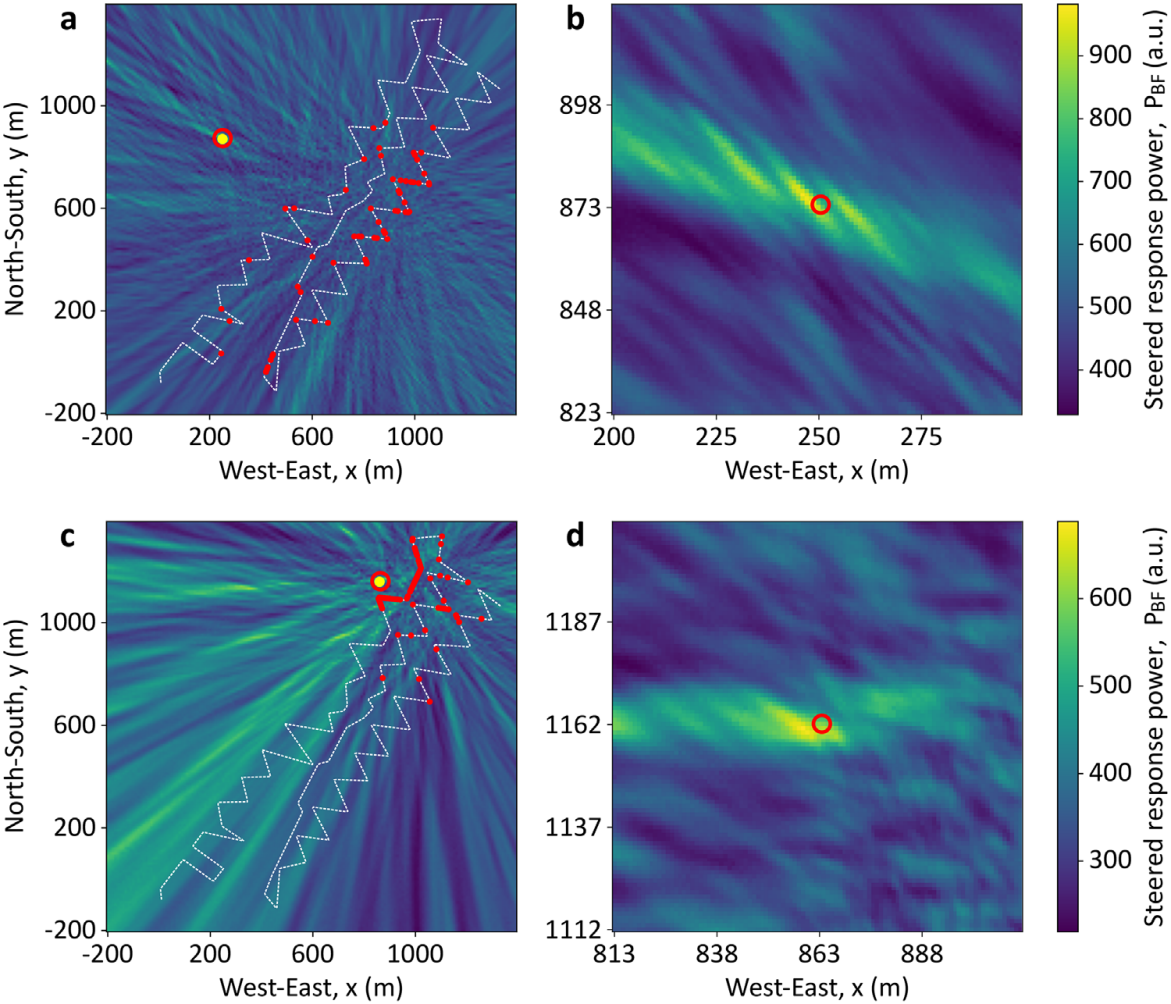
Acoustic images obtained with the best‐quality channels for sources T180 and T141. Acoustic images obtained using delay‐and‐sum beamforming for two acoustic sources. The 60 most reliable DAS channels used in each case are shown as red dots over the optical fiber (dashed white lines). a) Image obtained for the source T180 with b) a zoom‐in of the region with the highest steered response power of the entire image. c,d) correspond to the acoustic image obtained for the source T141 and its respective zoom‐in. The real source positions are indicated with red circles, while the brightest yellow pixels correspond to the estimated source locations. In addition, yellow dots, centered at the respective positions of the highest steered response power, have been added to a,b) to facilitate visualization.

**Figure** **4** presents acoustic images for two vibroseis truck positions, T180 and T141, using the proposed imaging approach (detailed in Experimental Section). Figure 4a shows the image for T180, created using the 60 most reliable DAS channels, selected through blind quality evaluation (see Experimental Section). Note that these channels, marked as red dots along the white dashed fiber path, form a sparse, unevenly distributed array. The brightest yellow region shows the area with the highest steered response power, indicating the estimated location of the acoustic source. Considering the small pixel size, a larger yellow dot is added to facilitate the visual localization of the detected source position, while the actual source location is marked with a red circle. The localization error is only 2.0 m, corresponding to a relative error of 0.37% with respect to the fiber centroid (537.4 m from the source) and 0.56% with respect to the nearest fiber position (358.6 m from the source), demonstrating high accuracy of the imaging method. Figure 4b zooms in to show the close match between the estimated (brightest yellow pixels) and actual (red circle) positions of the vibroseis track. The estimated acoustic velocity is 343.0 m/s.

Figure 4c,d shows similar results for T141, where the source is closer to the fiber. The estimated velocity is 327.0 m s^−1^ in this case, confirming local velocity variations as noted in the PoroTomo study.^[^ ^39^, ^40^ ^]^ Despite the proximity, the error is 4.0 m, i.e., larger than T180, and corresponding to a relative error of 0.66% with respect to the fiber centroid (604.9 m from the source) and 6.27% with respect to the nearest fiber position (63.7 m from the source). This larger error can be attributed to the smaller array aperture for the source T141, where the selected channels are clustered near the source, leading to a smaller aperture and reduced spatial resolution. This effect is particularly evident at greater distances from the selected DAS channels, where elongated patterns converging toward the centroid of the array become more noticeable in Figure 4c. The larger error could also be attributed to the weaker signals measured by the selected channels, likely caused by the poor strain coupling between the soil and the optical fiber section where these channels are positioned. Figure S2 (Supporting Information) confirms this via lower average reliability scores (β_*i*_) for T141, compared to the higher, more evenly distributed β_*i*_ values for T180. This results in better image contrast and localization accuracy for T180 (see color scale differences in Figure 4). These results demonstrate the effectiveness of the proposed blind channel selection approach for DAS‐based near‐field acoustic images and source localization.

**Figure 5:**
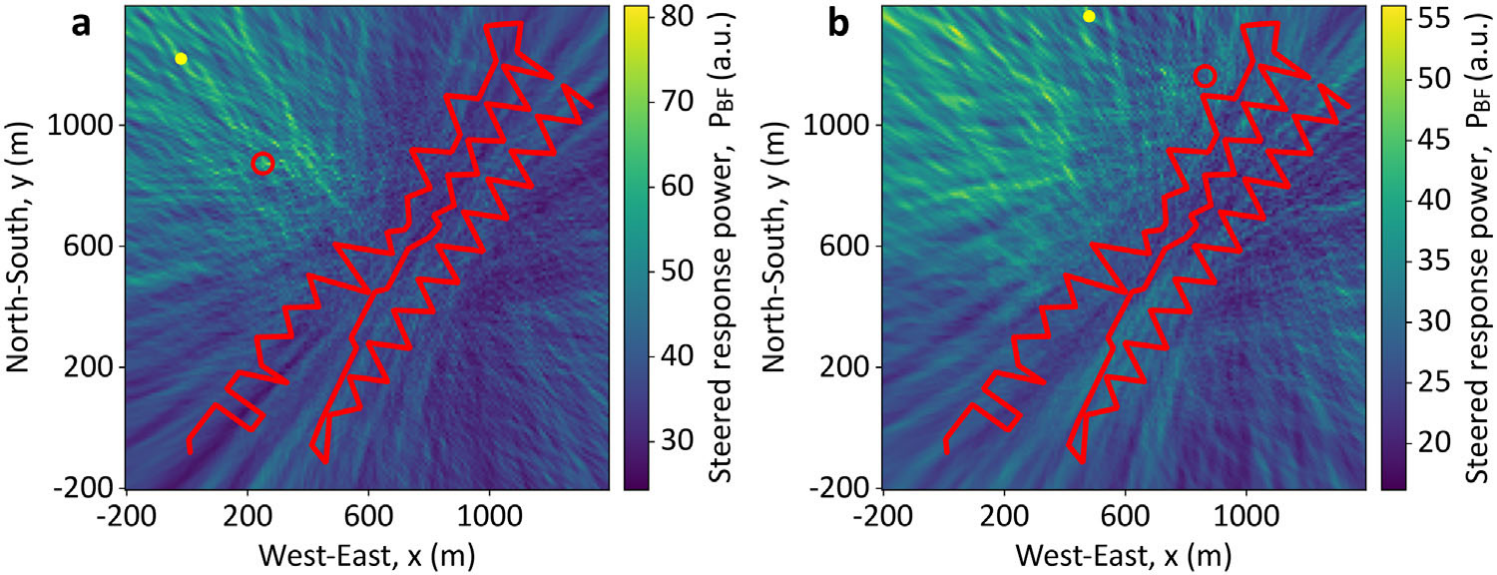
Acoustic images obtained with all DAS channels for T180 and T141. Acoustic images obtained using delay‐and‐sum beamforming for the acoustic source a) T180 and b) T141, using all 863 DAS channels (red dots) over the sensing fiber. The real source positions are indicated with red circles, while the brightest yellow pixels correspond to the estimated source locations. In addition, yellow dots, centered at the respective positions of the highest steered response power, have been added to facilitate visualization. It is important to note the lower steered response power in this case compared to Figure 4.

To further highlight the impact of the DAS channel selection, **Figure** **5** shows acoustic images using all 863 DAS channels without applying any channel quality selection. Results clearly demonstrate that including low‐quality channels in the processing significantly reduces the maximum steered response power (compare the colormap scales in Figures 4 and 5), impairing the accuracy of the acoustic images and acoustic source localization. As a result, the estimation errors substantially increase to 440 m for T180 and 431 m for T141, corresponding to 74.4% and 71.3% relative errors, respectively, compared to the distances between the sources and optical fiber centroid. The obtained acoustic images provide only a vague indication that the source is located somewhere to the northwest of the fiber, highlighting the importance of excluding low‐quality channels for accurate DAS‐based acoustic imaging and source localization.

### 2.4 Depth Resolution

**Figure 6:**
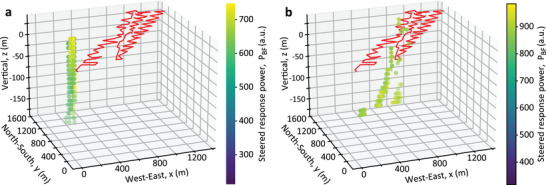
Thresholded 3D acoustic images obtained for sources T180 and T141. 3D imaging applied to experiments a) T180 and b) T141 on a Cartesian grid with 10 m spacing in each coordinate. A threshold is applied to the output of the delay‐and‐sum processing to display only the coordinates with the highest steered response power, thereby improving the visualization of the results in three dimensions.

**Figure 7:**
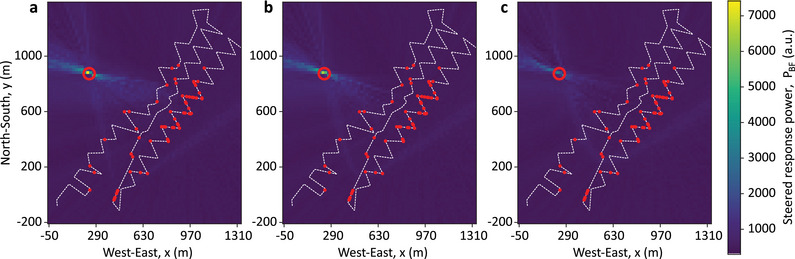
Acoustic images obtained from a simulated mechanical wave propagation for three vertical positions. Simulation of the wave propagation with an acoustic source located at the same position as T180, with a vertical position of *z* = 15 m. The acoustic images are obtained using delay‐and‐sum with the 60 most reliable DAS channels selected from the experimental data in Figure 5, for vertical positions of a) *z*  =  15 m, b) *z*  =   − 80 m, and c) *z*  =   − 160 m. The low depth resolution can be attributed to the limited spatial extension of the DAS array in the vertical direction, resulting in poor vertical spatial resolution.

This section aims to demonstrate that true 3D imaging is not feasible in this case due to the predominantly planar layout of the optical fiber used in this experiment. Although the optical fiber lies on a terrain with different elevations, the acoustic images in Figures 4 and 5 are generated at a constant *z*‐level, corresponding to the vertical centroid of the fiber. This simplification is necessary because the limited vertical extent of the fiber provides poor vertical spatial resolution, which restricts the ability of the DAS array to discriminate along the depth dimension. To assess the impact of this limitation, **Figure** **6** shows 3D beamforming results on an (*x*, *y*, *z*) grid, which is scanned with 10 m spacing for experiments T180 and T141. For clarity, only regions with the highest steered response around the source are displayed in this 3D image, obtained after thresholding the beamforming output. In Figure 6a (T180), strong responses appear even at depths like *z* = −160 m, centered around the same (*x*, *y*) location. A similar situation is observed for T141 in Figure 6b; however, in this case, the horizontal coordinates with high steered powers become scattered as depth increases. While reflections from underground layers could contribute to this response, the main cause is more likely due to the limited vertical resolution of the DAS array. To verify this, an acoustic source at the T180 location is simulated in a homogeneous medium (i.e., no reflections). **Figure** **7** shows acoustic images across three horizontal planes: *z* = 15 m (actual source level), *z* = −80 m, and *z* = −160 m. Despite no additional sources or scatterers, high steered responses persist at all depths, with only a slight reduction at *z*  = ‐160 m, even though the only simulated source is at z = 15 m. These results confirm that the nearly planar (2D) deployment of the sensing fiber in this case prevents accurate volumetric (3D) imaging and that reliable 3D reconstruction would require a deployment with sufficient vertical aperture and angular diversity. In the present case, vertical steering is therefore inefficient and computationally wasteful. More critically, failing to account for such geometric limitations can lead to false interpretations, incorrectly suggesting the presence of sources where none exist.

### 2.5 Dependence on the Acoustic Propagation Speed

**Figure 8:**
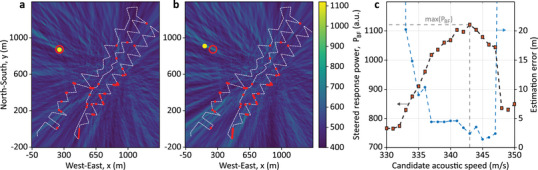
Effect of the acoustic wave velocity on the accuracy of the proposed imaging method. Acoustic images for experiment T180 obtained using the 60 most reliable DAS channels, with velocities slightly below and above the optimal 343 m s^−1^ (shown in Figure 4a): a) 338 m s^−1^, and b) 348 m s^−1^. c) Steered response power (red squares, left axis) and corresponding source localization error (blue circles, right axis) as a function of candidate acoustic velocities, illustrating the sensitivity of the imaging method to velocity variations in this case and the effectiveness of the blind velocity selection procedure.

The proposed imaging approach requires the acoustic propagation speed to convert the distance between each scanned point (pixel in the image) and the optical fiber into the corresponding time delays for each DAS channel. However, this parameter is often unknown, as in the present experiment. To address this, the method assumes a homogeneous but unknown velocity across the area and estimates the source position by finding the grid point (*x*, *y*) that maximizes the steered response power. This process is repeated over a range of candidate velocities representative of the experimental conditions.^[^ ^39^, ^40^ ^]^ **Figures** **8**a,b illustrate the impact of small velocity deviations (± 5 m s^−1^) on the imaging accuracy for experiment T180, compared with the optimal case at 343 m s^−1^ shown in Figure 4a. Results point out that even slight velocity errors noticeably degrade the imaging accuracy. This effect is quantified in Figure 8c, where both the steered response power (left axis) and localization error (right axis) are plotted against the candidate velocity. Results demonstrate that selecting the velocity that maximizes the steered response power reduces estimation errors (right axis) compared to the true source position.

## 3 Discussion

**Figure 9:**
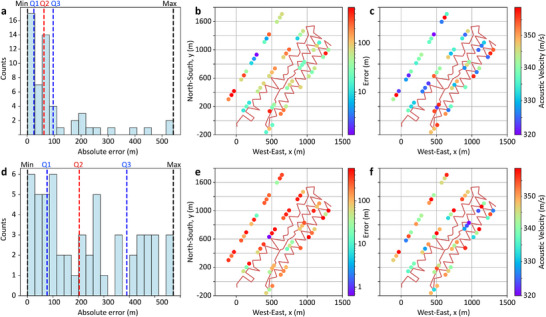
Estimation of acoustic source locations and propagation velocities for the 55 source locations. a) Histogram of the absolute errors between estimated and actual source locations, indicating minimum (1.33 m), first quartile (Q1: 24.61 m), median (Q2: 62.05 m), third quartile (Q3: 96.25 m), and maximum (541.82 m) values using the best 60 DAS channels. b) Spatial distribution of absolute errors for each source with a colormap indicating the error level. c) Estimated propagation velocity for each source. Figures d–f) repeat the same analysis but using all 863 DAS channels, indicating minimum (0.64 m), first quartile (Q1: 77.11 m), median (Q2: 203.60 m), third quartile (Q3: 390.19 m), and maximum (572.70 m) values.

This proof‐of‐concept demonstrates that blind delay‐and‐sum, although being the simplest beamforming method, can generate accurate acoustic images based on DAS measurements. Results show that the image quality can be strongly affected by local wave propagation characteristics, fiber orientation, and DAS directional sensitivity, especially over large arrays. Based on these limitations, we evaluate the localization performance of the method by producing acoustic images for 55 vibroseis source positions. **Figure** **9**a presents a histogram of the absolute localization errors for the 55 source positions, verifying a minimum of 1.33 m and a median of 62.05 m. The spatial distribution of these errors is shown in Figure 9b, where each acoustic source is color‐coded according to its absolute estimation error. Note that the estimation error is not determined solely by the source‐fiber distance, as would be expected under ideal conditions, but is also affected by factors determining the local propagation of the mechanical wave and its angle of incidence on the sensing fiber. Indeed, sources closer to the DAS array centroid do not always yield smaller errors, while some distant sources achieve better accuracy. This counterintuitive behavior can also be attributed to the reduced spatial resolution caused by the clustering of selected DAS channels when the source is near the array, as seen in Figure 4.

Figure S3 (Supporting Information) also illustrates this effect using four sources at different positions, all with absolute errors near the median. For nearby sources (Figure S3a,b, Supporting Information), reliable channels cluster around the source, reducing the DAS array aperture and the spatial resolution. In contrast, for distant sources (Figure S3c,d, Supporting Information), channels are more scattered, improving resolution despite weaker acoustic signals reaching the fiber, yielding comparable errors. Figure 9c illustrates the spatial distribution of the estimated velocities, showing well‐estimated source clusters, like triplets (T179, T180, T181) and (T122, T123, T124), consistent with known local acoustic velocity variations.^[^ ^39^, ^40^ ^]^ Figures 9d and 8f repeat the analysis using all 863 channels, showing degraded imaging and localization, confirming the effectiveness of the proposed blind channel selection. Overall, selecting only the highest‐quality channels significantly enhances DAS‐based beamforming and acoustic imaging precision.

In this first demonstration, generating a single acoustic image takes ≈22.5 s using Python on a desktop with an Intel i7‐11700 CPU @ 2.50 GHz. Since 40 images (each with a different acoustic velocity) are required, the total processing time is ≈15 minutes. However, current CPU usage is only ≈20%, suggesting a five‐fold speedup via parallelization and reducing the image generation to ≈4.5 s and the total time to ≈3 min. Calculating phase cross‐correlations for 863 channels and computing quality indicators adds ≈10 min, performed via a basic CUDA implementation using fast Fourier transforms on a GeForce GTX1060 GPU (6 GB). This remains the main bottleneck. Ideally, the total processing time should remain under the 20 s temporal window used for the steered response power estimation. Optimizing GPU code^[^ ^48^ ^]^ and using advanced parallel techniques could greatly reduce the processing time, potentially enabling real‐time DAS‐based acoustic cameras^[^ ^49^, ^50^ ^]^ for acoustic video and moving‐source tracking.

This proof‐of‐concept study uses the basic delay‐and‐sum beamformer,^[^ ^24^, ^41^, ^42^ ^]^ leaving room for improvement through advanced techniques. More sophisticated methods, such as delay‐multiply‐and‐sum,^[^ ^51^, ^52^ ^]^ could enhance spatial resolution and contrast by applying weights to the selected DAS channels, albeit with higher computational cost. Optimizing the DAS listening beam based on the fiber layout is another promising direction. Techniques to narrow beams and reduce sidelobes could significantly improve image quality. Adaptive beamformers like minimum variance methods,^[^ ^53^, ^54^, ^55^, ^56^ ^]^ which apply dynamic weighting, are particularly suited for complex or changing environments.

A current limitation observed in this first demonstration is the reduced spatial resolution caused by DAS channels clustering when the acoustic source is near the fiber (Figure 4; Figure S3, Supporting Information). Using a broader channel distribution could improve array aperture but may degrade the image quality if lower‐quality channels are included. Sparse array processing^[^ ^57^, ^58^, ^59^, ^60^, ^61^ ^]^ offers potential solutions by optimizing sparse configurations with high‐quality channels, using techniques like minimum redundancy, co‐prime, nested, or super‐nested arrays.^[^ ^62^, ^63^, ^64^, ^65^ ^]^ Additionally, adaptive channel selection based on both signal quality and fiber orientation could improve accuracy by aligning DAS sensitivity with the expected wavefronts.

Since the beamforming performance depends on the propagation medium, environmental compensation methods^[^ ^66^, ^67^ ^]^ could be employed to account for spatial variations in the wave speed, attenuation, and dispersion, improving the image accuracy and potentially enabling estimation of emitted wave strengths. Although this study uses a time‐domain DAS with meter‐scale resolution, the method could be extended to frequency‐domain interrogations offering millimetric or centimetric resolution, enabling high‐frequency acoustic imaging. Together, these improvements could make DAS‐based acoustic imaging a powerful, versatile tool for applications ranging from geophysics and infrastructure monitoring to scenarios where conventional sensor arrays are impractical.

## 4 Experimental Section

### Digital Bandpass Filtering

The preprocessing begins by filtering the acoustic channels to reduce noise and enhance signal quality. The target signal lies between 5 and 80 Hz. Three filtering methods are tested in this work. First, two DAS‐specific filters were independently applied: an adaptive frequency filter^[^ ^68^ ^]^ and the self‐supervised deep learning model jDAS.^[^ ^69^ ^]^ However, both assume linear fiber layouts, so the fiber was segmented into straight sections to apply them. While the adaptive filter shows no clear benefits, jDAS introduces artifacts that disrupt the phase‐correlation‐based channel ranking. The third method uses a zero‐phase, fourth‐order Butterworth filter tailored to the signal spectral range. Among all, the Butterworth filter offers the best performance for the array processing techniques and therefore was adopted in this work.

### Evaluation of the Acoustic Channel Quality

To secure optimal beamforming performance, only DAS channels with high acoustic signal quality should be used. Since DAS channel quality varies randomly along the fiber due to orientation and source position,^[^ ^17^, ^26^ ^]^ a robust selection method was required. The variability further increases in anisotropic media, where reflections affect the wave propagation. A basic option was to assess the acoustic signal SNR, but this could be misleading in practice. Reflections and reverberations may artificially raise SNR while degrading signal purity.^[^ ^26^ ^]^ If the source waveform were known, one could directly compare measured and emitted signals to identify reliable channels. However, in most real applications, the waveform was unknown, so a blind channel selection approach was needed. A practical solution was to assess the waveform similarity between DAS channels using cross‐correlation. Low‐quality DAS channels typically exhibit poor correlation with others, while high‐quality ones correlate well with those measuring the same acoustic wave. Traditional amplitude‐based correlation, however, was biased by strong signal sections. A better approach was the use of phase cross‐correlation,^[^ ^45^ ^]^ which compares the instantaneous phase and was insensitive to amplitude variations. Since DAS provides real‐valued signals *x*(*t*), the analytical signal *x_a_* (*t*) was first computed using Hilbert transform:^[^ ^70^ ^]^

(1) $$\begin{array}{c} x_{a}\left(t\right)=F^{-1}\left\{X\left(f\right)\cdot 2\mu \left(f\right)\right\}=x\left(t\right)*\left(\delta \left(t\right)+\frac{j}{\pi t}\right)\\ =x\left(t\right)+j\left(\frac{j}{\pi t}*x\left(t\right)\right)=x\left(t\right)+jH\left\{x\left(t\right)\right\}\\ \# \end{array}$$

where $H\left\{x(t)\right\}=\hat{x}\left(t\right)$ is the Hilbert transform of the DAS measurement *x*(*t*) at a given fiber position, μ(*t*) is the Heaviside step function, δ(*t*) is the Dirac‐delta function, *F* ^−1^{·} is the inverse Fourier transform operator, * represents the convolution o00perator, and *X*(*f*) is the Fourier transform of *x*(*t*). As its name suggests, the phase cross‐correlation uses the phase *e* ^*jb*(*t*)^ of the analytic signal $x_{a}\left(t\right)=x\left(t\right)+j\hat{x}\left(t\right)=a\left(t\right)e^{jb(t)}$ for the calculation. For a pair of discrete‐time real‐valued signals *x*_1_[*k*] and *x*_2_[*k*], representing the sampled acoustic waveforms at two fiber positions, the PCCF is then defined as follows:

(2) $$\mathrm{PCCF}\left[n\right]=R\left\{\frac{1}{N}\left(e^{jb_{1}\left[k\right]}⊗e^{jb_{2}\left[k\right]}\right)\right\}=R\left\{\frac{1}{N}\sum_{n=1}^{N}e^{-jb_{1}\left[k\right]}e^{jb_{2}\left[k+n\right]}\right\}$$

where ⊗ represents the cross‐correlation operator, $e^{jb_{1}\left[k\right]}$ and $e^{jb_{2}\left[k\right]}$ are the respective phases of the analytical signals of *x*_1_[*k*] and *x*_2_[*k*], *R*{·} denotes the real‐part operator, and *N* is the number of temporal samples. The magnitude of the analytical signal corresponds to the amplitude envelope of the real‐valued DAS signal.^[^ ^70^ ^]^ To compute the PCCF, the cross‐correlation was performed between two analytical signals normalized by their envelopes, *a*(*t*), ensuring phase comparison was not biased by amplitude variations. In this case, the PCCF was efficiently calculated via fast Fourier transforms using Python's SciPy library, specifically the *hilbert* and *correlate* functions.

PCCF is then used to compute a local similarity indicator, κ_*ij*_, between channels *i* and *j*, defined as the ratio of the PCCF peak to the RMS of a surrounding window *W*:

(3) $$\kappa_{ij}=\frac{\max(\mathrm{PCC}F_{ij})}{\mathrm{RMS}\left(W\right)}$$

with $\mathrm{RMS}\left(W\right)=\sqrt{\frac{1}{2L}\sum {\mathrm{PCCF}}_{ij}^{2}\left(W\right)}$, where the sum is computed over a 2*L*‐sample window *W* centered on PCCF peak, and excluding the peak itself. Here *L* = 2000 samples, which is equivalent to 2 s at the sampling frequency *f_s_* = 1000 Hz.

Unlike previous definitions,^[^ ^26^ ^]^ the maximum (not the absolute maximum) of the PCCF is used to reduce scores for counter‐phase channels, improving beamformer performance (see Supporting Information 1). A low κ_*ij*_ indicates poor correlation, echoes from multipath propagation, or narrowband signal periodicity. In contrast, a high κ_*ij*_ reflects a strong, isolated correlation peak, indicating a reliable broadband waveform with minimal distortion or noise. This makes κ_*ij*_ effective for identifying high‐quality DAS channel pairs.

The local similarity indicator κ_*ij*_ forms a symmetric *M* × *M* matrix, since κ_*ij*_ = κ_*ji*_. To reduce computation time, only the upper (or lower) triangular portion, including the diagonal, is calculated. Despite this optimization, the method retains a quadratic time complexity of *O*(*M* ^2^), making it impractical to process all 8630 original channels in this case.

After calculating the κ_*ij*_ matrix, the global reliability indicator β_*i*_ is calculated as the RMS of the 1 × *M* vector **κ**_*i*_ =  [κ_*i*,1_,  κ_*i*,2_,..,  κ_*i*,*M*_], excluding *j* = *i*:

(4) $$\beta_{i}=\sqrt{\frac{1}{M}\sum_{j=1,\;\;j\neq i}^{M}\kappa_{ij}^{2}\;}$$

The global score β_*i*_ quantifies the quality of the *i*‐th DAS channel, allowing all channels to be ranked and the highest‐quality ones selected for beamforming‐based acoustic imaging. In this case, the optimal number of DAS channels was determined through a preliminary analysis using different numbers of DAS channels and source positions, followed by a statistical evaluation (see Supporting Information 4) to identify the condition that minimizes the average localization error across the imaged area.

### Acoustic Imaging Using Delay‐and‐Sum Beamforming

The proposed method generates acoustic images in the near‐field region defined by the fiber layout, where the wavefronts are assumed spherical (point source model). This near‐field is bounded by the Fraunhofer distance, *d_f_* = 2*D* ^2^/λ, with *D* as the array maximum length and λ the acoustic wavelength in the medium.^[^ ^23^, ^24^, ^25^ ^]^ Under the most restrictive conditions (lowest acoustic frequencies and highest propagation velocities),^[^ ^39^, ^40^ ^]^ this distance was ≈62.5 km, well beyond the imaged area, thereby justifying the spherical wave assumption.

To create a visual acoustic map, the near‐field region is divided into a Cartesian grid (*x*,  *y*). Each pixel is treated as a potential acoustic source *s_j_* (*t*), with $j=1,\text{…},P_{x}\cdot P_{y}$, where *P_x_* and *P_y_* are the number of grid points in *x* and *y*. The signals *y_i_* (*t*) measured by *M* DAS channels (i=1,…,M) can be expressed as:^[^ ^71^ ^]^

(5) $$\left[\begin{array}{c} \begin{array}{c} y_{1}\left(t\right)\\ y_{2}\left(t\right) \end{array}\\ \vdots \\ y_{M}\left(t\right) \end{array}\right]=\left[\begin{array}{c} \begin{array}{cc} \begin{array}{cc} a_{1}\left(x,y\right) & a_{2}\left(x,y\right) \end{array} & \begin{array}{cc} \text{…} & a_{P_{x}\cdot P_{y}}\left(x,y\right) \end{array} \end{array} \end{array}\right]\left[\begin{array}{c} \begin{array}{c} s_{1}\left(t\right)\\ s_{2}\left(t\right) \end{array}\\ \vdots \\ s_{P_{x}\cdot P_{y}}\left(t\right) \end{array}\right]+\left[\begin{array}{c} \begin{array}{c} n_{1}\left(t\right)\\ n_{2}\left(t\right) \end{array}\\ \vdots \\ n_{M}\left(t\right) \end{array}\right]$$

where *n_i_* (*t*) (for i=1,…,M) represents the measurement noise in each DAS channel, while the *M* × 1 steering vectors *a_j_* (*x*,*y*) (for $j=1,\text{…},P_{x}\cdot P_{y}$) depend on the array geometry, the (*x*, *y*) location, and the propagation properties of the medium. These complex‐valued vectors account for attenuation and delays as waves travel from each potential source location (pixel) to the fiber.

The proposed imaging method can be viewed as estimating each emitted acoustic signal *s_j_* (*t*) by inverting the steering matrix and applying it to the DAS measurements *y_i_* (*t*), the basis of delay‐and‐sum beamforming.^[^ ^24^, ^41^, ^42^ ^]^ Unlike conventional beamforming, which aligns signals to focus on a given source, the approach scans all grid cells across a large area, regardless of source presence. This enables the generation of acoustic images covering the entire 360° space around the sensing optical fiber, thereby enabling the potential detection of multiple acoustic sources.

Among various acoustic imaging algorithms,^[^ ^43^, ^72^ ^]^ as a proof‐of‐concept we adopt the simplest: delay‐and‐sum beamforming.^[^ ^24^, ^41^, ^42^ ^]^ This technique delays and weights the signals from each DAS channel to steer the beam to a specific (*x*, *y*) location. The output *z*(*t*, *x*, *y*) estimates the *s_j_* (*t*) at that location by summing time‐shifted, weighted signals:^[^ ^24^, ^41^, ^42^ ^]^

(6) $$z\left(t,x,y\right)=\sum_{i=1}^{M}w_{i}y_{i}\left(t-\Delta \tau_{i}\right)$$

where Δτ_*i*_ is the relative delay and *w_i_* is the weight applied to the *i*‐th DAS channel.

This process is repeated over all (*x*, *y*) positions in the imaged area. In delay‐and‐sum beamforming, weights *w_i_* are typically uniform and normalized as *w_i_* = 1/*M*​ to ensure that $\sum_{i=1}^{M}w_{i}=1$. Applying relative time delays compensates for propagation delays from the spherical wavefront (defined by steering vectors), aligning signals in phase from the target location. Summing up these in‐phase signals strengthens the beamformer output, while signals from other locations interfere destructively and attenuate. This acts as a spatial filter, enhancing signals from the focused point (*x*, *y*) and suppressing others. Then, the amplitude of each image pixel is associated to the local steered response power *P_BF_* (*x*,*y*), defined as:

(7) $$P_{BF}\left(x,y\right)=\int {\left(z\left(t,x,y\right)\right)}^{2}dt=\int {\left(\sum_{i=1}^{M}w_{i}y_{i}\left(t-\Delta \tau_{i}\right)\right)}^{2}dt$$

where Δτ_*i*_ is a multiple of the sampling interval in the discrete‐time domain.

Finally, the resulting acoustic image is obtained by coloring each pixel proportional to the local steered response power *P_BF_* (*x*,*y*).

## Conflict of Interest

The authors declare no conflict of interest.

## Supporting information



Supporting Information

## Data Availability

The data that support the findings of this study are openly available in oroTomo Natural Laboratory Horizontal and Vertical Distributed Acoustic Sensing Data at https://dx.doi.org/10.15121/1778858, reference number 6760.

## References

[1] A. H. Hartog , Introduction to Distributed Optical Fiber Sensors, CRC Press, Boca Raton, FL 2017.

[2] J. Li , M. Zhang , Light Sci. Appl. 2022, 11, 128. 35525847 10.1038/s41377-022-00811-x PMC9079107

[3] M. A. Soto , F. Di Pasquale , in Handbook of Optical Fibers (Ed: G. D. Peng ), Springer, Singapore 2018.

[4] A. Motil , A. Bergman , M. Tur , Opt. Laser Technol. 2016, 78, 81.

[5] M. A. Soto , in Handbook of Optical Fibers ( G. D. Peng ), Springer, Singapore 2018.

[6] T. Horiguchi , K. Shimizu , T. Kurashima , M. Tateda , Y. Koyamada , J. Lightwave Technol. 1995, 13, 1296.

[7] J. C. Juarez , E. W. Maier , K. N. Choi , H. F. Taylor , J. Lightwave Technol. 2005, 23, 2081.

[8] L. Palmieri , L. Schenato , Open Opt. J. 2013, 7, 104.

[9] X. Fan , in Handbook of Optical Fibers (Ed: G. D. Peng ), Springer, Singapore 2018.

[10] Z. He , Q. Liu , J Lightwave Technology 2021, 39, 3671.

[11] Y. Shang , M. Sun , C. Wang , J. Yang , Y. Du , J. Yi , W. Zhao , Y. Wang , Y. Zhao , J. Ni , Sensors 2022, 22, 6060. 36015819 10.3390/s22166060 PMC9412507

[12] G. P. Agrawal , Nonlinear Fiber Optics, 5th ed. Academic Press, San Diego, CA 2013.

[13] R. W. Boyd , Nonlinear Optics, 2nd ed., Academic Press, San Diego, CA, London 2003.

[14] K. I. Aoyama , K. Nakagawa , T. Itoh , IEEE J. Quantum Electron. 1981, 17, 862.

[15] W. Eickhoff , R. Ulrich , Appl. Phys. Lett. 1981, 39, 693.

[16] L. Schenato , L. Palmieri , M. Camporese , S. Bersan , S. Cola , A. Pasuto , A. Galtarossa , P. Salandin , P. Simonini , Sci. Rep. 2017, 7, 14686. 29089632 10.1038/s41598-017-12610-1 PMC5665914

[17] G. Fang , Y. E. Li , Y. Zhao , E. R. Martin , Geophys. Res. Lett. 2020, 47, 2019GL086115.

[18] M. R. Fernández‐Ruiz , M. A. Soto , E. F. Williams , S. Martin‐Lopez , Z. Zhan , M. Gonzalez‐Herraez , H. F. Martins , APL Photonics 2020, 5, 030901.

[19] M. R. Fernández‐Ruiz , H. F. Martins , E. F. Williams , C. Becerril , R. Magalhães , L. Costa , S. Martin‐Lopez , Z. Jia , Z. Zhan , M. González‐Herráez , J. Lightwave Technol. 2022, 40, 1453.

[20] F. Huot , A. Lellouch , P. Given , B. Luo , R. G. Clapp , T. Nemeth , K. T. Nihei , B. L. Biondi , Seismol. Soc. Am. 2022, 93, 2543.

[21] F. Staněk , G. Jin , J. Simmons , Front. Earth Sci. 2022, 10, 907749.

[22] A. Lellouch , R. Schultz , N. J. Lindsey , B. L. Biondi , W. L. Ellsworth , J. Geophys. Res.: Solid Earth 2021, 126, 2020JB020462.

[23] J. Benesty , J. Chen , Y. Huang , Microphone Array Signal Processing, Springer, New York 2008.

[24] D. H. Johnson , D. E. Dudgeon , Array Signal Processing: Concepts and Techniques, Pearson, Upper Saddle River, New Jersey, USA 1993.

[25] W. Liu , S. Weiss , Wideband Beamforming: Concepts and Techniques, Wiley, New York 2010.

[26] F. Muñoz , M. A. Soto , Nat. Commun. 2022, 13, 4019. 35821369 10.1038/s41467-022-31681-x PMC9276755

[27] R. Xu , J. Sun , Y. Wang , S. Zhang , W. Zhong , Z. Wang , IEEE Sens. J. 2023, 23, 11656.

[28] E. M. Ku , G. L. Duckworth , Proc. Mtgs. Acoust. 2013, 19, 070053.

[29] N. J. Lindsey , E. R. Martin , D. S. Dreger , B. Freifeld , S. Cole , S. R. James , J. B. Ajo‐Franklin , Geophys. Res. Lett. 2017, 44, 11.

[30] N. J. Lindsey , T. C. Dawe , J. B. Ajo‐Franklin , Science 2019, 366, 1103. 31780553 10.1126/science.aay5881

[31] M. P. A. van den Ende , J. P. Ampuero , Solid Earth 2021, 12, 915.

[32] D. Badillo , M. A. Soto , in Optica Sensing Congress 2024 (AIS, LACSEA, Sensors, QSM), Technical Digest Series , Optica Publishing Group, Toulouse, France 2024.

[33] B. Lu , B. Wu , J. Gu , J. Yang , K. Gao , Z. Wang , L. Ye , Q. Ye , R. Qu , X. Chen , H. Cai , Opt. Express 2021, 29, 3147. 33770920 10.1364/OE.414598

[34] P. Stoica , A. Nehorai , IEEE Trans. Acoust. Speech Signal Process. 1989, 37, 720.

[35] L. Jiajing , W. Zhaoyong , L. Bin , W. Xiao , L. Luchuan , Y. Qing , Q. Ronghui , C. Haiwen , Opt. Lett. 2019, 44, 1690. 30933123 10.1364/OL.44.001690

[36] N. Shpalensky , L. Shiloh , H. Gabai , A. Eyal , Opt. Express 2018, 26, 17690. 30119579 10.1364/OE.26.017690

[37] J. Chen , H. Li , K. Ai , S. Zhu , C. Fan , Z. Yan , Q. Sun , IEEE Trans. Instrum. Meas. 2025, 74, 9504311.

[38] A. Manikas , C. Proukakis , IEEE Trans. Signal Process 1998, 46, 2166.

[39] K. L. Feigl , L. M. Parker , PoroTomo Final Technical Report: Poroelastic Tomography by Adjoint Inverse Modeling of Data from Seismology, Geodesy, and Hydrology, University of Wisconsin–Madison, Madison, Wisconsin, USA 2019.

[40] K. L. Feigl , in 43rd Workshop on Geothermal Reservoir Engineering , Stanford University, Stanford, USA 2018, p.1715.

[41] V. Perrot , M. Polichetti , F. Varray , D. Garcia , Ultrasonics 2021, 111, 106309. 33360053 10.1016/j.ultras.2020.106309

[42] A. Cigada , F. Ripamonti , M. Vanali , Mech. Syst. Signal Process. 2007, 21, 2645.

[43] R. Merino‐Martínez , P. Sijtsma , M. Snellen , T. Ahlefeldt , J. Antoni , C. J. Bahr , D. Blacodon , D. Ernst , A. Finez , S. Funke , T. F. Geyer , S. Haxter , G. Herold , X. Huang , W. M. Humphreys , Q. Leclère , A. Malgoezar , U. Michel , T. Padois , A. Pereira , C. Picard , E. Sarradj , H. Siller , D. G. Simons , C. Spehr , CEAS Aeronaut J. 2019, 10, 197.

[44] X. Zeng , C. H. Thurber , Y. Luo , E. Matzel , in 42nd Workshop on Geothermal Reservoir Engineering , Stanford University, Stanford, California, USA 2017, p. 212.

[45] M. Schimmel , Bull. Seismol. Soc. Am. 1999, 89, 1366.

[46] M. Schimmel , E. Stutzmann , J. Gallart , Geophys. J. Int. 2011, 184, 494.

[47] S. Ventosa , M. Schimmel , L. Stutzmann , Seismol. Res. Lett. 2019, 90, 1663.

[48] P. J. Lu , H. Oki , C. A. Frey , G. E. Chamitoff , L. Chiao , E. M. Fincke , C. M. Foale , S. H. Magnus , W. S. McArthur , D. M. Tani , P. A. Whitson , J. N. Williams , W. V. Meyer , R. J. Sicker , B. J. Au , M. Christiansen , A. B. Schofield , D. A. Weitz , J. Real‐Time Image Proc. 2010, 5, 179.

[49] C. Zimmermann , C. Studer , in Proc. of 2010 IEEE International Symposium on Circuits and Systems , IEEE, Paris, France 2010, pp. 1419‐1419.

[50] M. M. Erić , in 2011 19thTelecommunications Forum (TELFOR) Proc. of Papers , IEEE, Belgrade, Serbia 2011, pp. 1036–1039.

[51] S. Paul , S. Mulani , N. Daimary , M. S. Singh , IEEE Trans. Instrum. Meas. 2022, 71, 1.

[52] L. Chen , Z. Liu , Z. Zhang , Y. Zhu , X. Liu , J. Hu , C. He , Mech. Syst. Signal Process. 2025, 224, 112206.

[53] R. G. Lorenz , S. P. Boyd , IEEE Trans. Signal Process. 2005, 53, 1684.

[54] M. Wax , Y. Anu , IEEE Trans. Signal Process. 1996, 44, 928.

[55] B. Luijten , R. Cohen , F. J. de Bruijn , H. A. W. Schmeitz , M. Mischi , Y. C. Eldar , R. J. G. van Sloun , IEEE Trans Med Imaging 2020, 39, 3967. 32746139 10.1109/TMI.2020.3008537

[56] Y. H. Choi , Digit. Signal Process. 2024, 146, 104370.

[57] X. Wang , M. Amin , X. Cao , IEEE Trans. Signal Process. 2018, 66, 340.

[58] S. A. Hamza , M. G. Amin , IEEE Trans. Signal Process. 2019, 67, 6215.

[59] Z. Zheng , Y. Fu , W. Q. Wang , IEEE Trans. Antennas Propag. 2021, 69, 2628.

[60] W. Peng , T. Gu , Y. Zhuang , Z. He , C. Han , Digit. Signal Process. 2022, 128, 103632.

[61] R. Cohen , Y. C. Eldar , IEEE Trans. Signal Process. 2020, 68, 4797.

[62] A. Moffet , IEEE Trans. Antennas Propag. 1968, 16, 172.

[63] P. Pal , P. P. Vaidyanathan , IEEE Trans. Signal Process. 2010, 58, 4167.

[64] C. L. Liu , P. P. Vaidyanathan , IEEE Trans. Signal Process. 2016, 64, 3997.

[65] P. P. Vaidyanathan , P. Pal , IEEE Trans. Signal Process. 2011, 59, 573.

[66] J. E. Michaels , in Structural Health Monitoring (SHM) in Aerospace Structures (Ed: F.‐G. Yuan ), Woodhead Publishing, Cambridge, UK 2016, pp. 255–284.

[67] P. C. Ostiguy , A. Le Duff , N. Quaegebeur , L. P. Brault , P. Masson , Struct. Health Monit. 2014, 13, 525.

[68] M. P. Isken , S. Heimann , C. Wollin , H. Bathke , T. Dahm , Lightguide – Seismological Tools for DAS Data, GFZ German Research Centre for Geosciences, Potsdam, Germany 2022.

[69] M. P. A. van den Ende , I. Lior , J. P. Ampuero , A. Sladen , A. Ferrari , C. Richard , IEEE Trans. Neural Netw. Learn. Syst. 2023, 34, 3371. 34919525 10.1109/TNNLS.2021.3132832

[70] J. O. Smith , Mathematics of the Discrete Fourier Transform (DFT): With Audio Applications, 2nd ed., W3K Publishing, Stanford, California, USA 2007.

[71] R. Schmidt , IEEE Trans. Antennas Propag. 1986, 34, 276.

[72] Q. Leclère , A. Pereira , C. Bailly , J. Antoni , C. Picard , , Int. J. Aeroacoust. 2017, 16, 431.

