# Supplementary material for: Near‐Field Acoustic Imaging Using Fiber‐Optic Distributed Acoustic Sensing and Beamforming Techniques
Source: Adv Sci (Weinh). 2025 Nov 14;13(6):e12513. doi: 10.1002/advs.202512513 (PMC12866732; doi:10.1002/advs.202512513)
Supplement: Supplementary file 1 — Supporting Information [file ADVS-13-e12513-s001.pdf]

Supporting Information

Near-Field Acoustic Imaging Using Fiber-Optic Distributed Acoustic Sensing and Beamforming Techniques

Marcelo A. Soto and Diego Badillo*
Department of Electronics Engineering, Universidad Técnica Federico Santa María, 2390123 Valparaíso, Chile
* E-mail: marcelo.sotoh@usm.cl

Supporting Information 1: Temporal waveforms from different DAS channels

The method proposed in this work to evaluate the quality of DAS channels corresponds to a variation of the blind reliability channel sorting method previously used for single-source localization using triangulation, where the local similarity indicator κ_{ij} was defined in terms of the absolute value of the phase cross-correlation function (PCCF). However, this study shows that for delay-and-sum beamforming, removing the calculation of the absolute value yields better results by assigning lower reliability scores to channels that are in counter-phase, either due to the intrinsic limitations of strain/strain-rate DAS measurements or by spatial wave properties. Consequently, in this work we redefine the local similarity indicator κ_{ij} in Equation (3) of the main text of the manuscript, while the global reliability score $\boldsymbol{\beta}_i$, defined in Equation (4), remains unchanged but uses the newly defined κ_{ij} values.

Figure S1 shows an example for the experiment T181, displaying measurements from the DAS channels corresponding to the eighth linear section of the sensing fiber. Waveforms are time-shifted based on the known location of the acoustic source, using differential distances scaled by the estimated average propagation speed. As a visual reference, the DAS channels plotted in blue represent 2 of the 60 best channels selected for beamforming in this case. Two different time intervals are plotted in the figure. In the first interval, channels 86 to 88 are observed to be in counter-phase relative to the two selected channels in red. Summing these channels as they are steered to the actual source results in a destructive summation and reduced steered response power. In the second interval, the two blue-selected channels still add in phase, exhibiting

consistent behavior, whereas the channels 86 to 88 show inconsistent behavior between both time intervals. Our objective is to sort DAS measurements so that selecting the m best channels secures constructive summation across the entire time window when the steering beam focuses on the acoustic source.

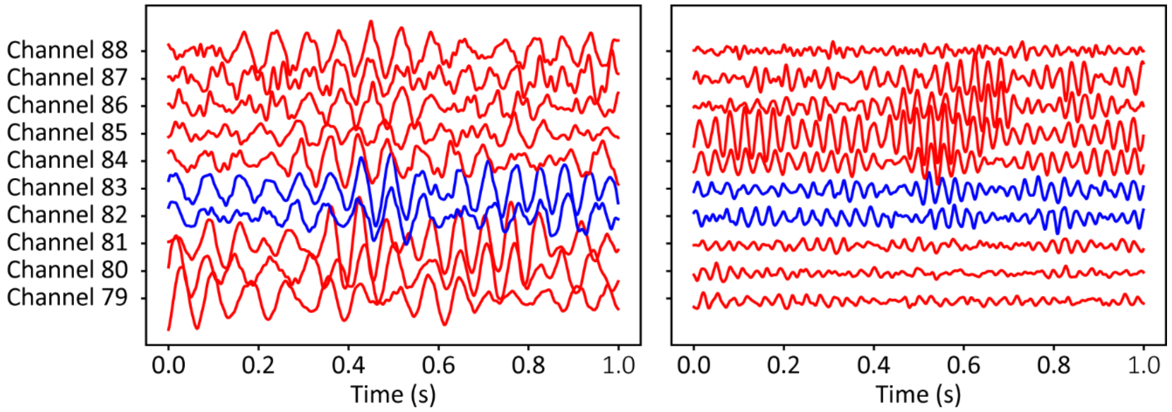

Figure S1. Acoustic waveforms in a linear section of the fiber corresponding to channels 79 to 88 for the acoustic source T181. Two one-second time intervals of data, measured at 2 s and 7 s after the start of the vibration are shown. These waveforms are time-shifted to point the array toward the source, assuming its location is known. Channels 82 and 83 (blue lines) correspond to high-quality channels selected by the proposed channel evaluation and selection method.

This analysis suggests that using the absolute value of the PCCF to define the local similarity indicator κ_{ij} is inappropriate in the context of beamforming-based acoustic imaging. This approach can compromise the steered response power associated with different regions of the imaged space. By removing the absolute value of the PCCF in the calculation of κ_{ij}, the method provides a better evaluation and ranking of the DAS channels quality, resulting in reduced source localization errors and improved imaging precision.

Supporting Information 2: Spatial distribution of the global reliability indicator $\boldsymbol{\beta_i}$

As described in the Methods section, the channel sorting method is designed to blindly exclude DAS channels that could detrimentally affect the array processing performance. A DAS channel might exhibit low SNR measurements if the vibration source is located such that the directivity of the sensing fiber towards the source is low. If this were the only reason for low SNR, then each channel should have, on average across different source locations, around the same

reliability score β_i. This is, however, not the case, as shown in Figure S2, which demonstrates that some sections of the sensing fiber consistently have lower reliability scores on average across all 55 analyzed acoustic source positions. This consistent low quality of some DAS channels could be attributed to the poor local strain coupling between the optical fiber and the ground in those respective regions.

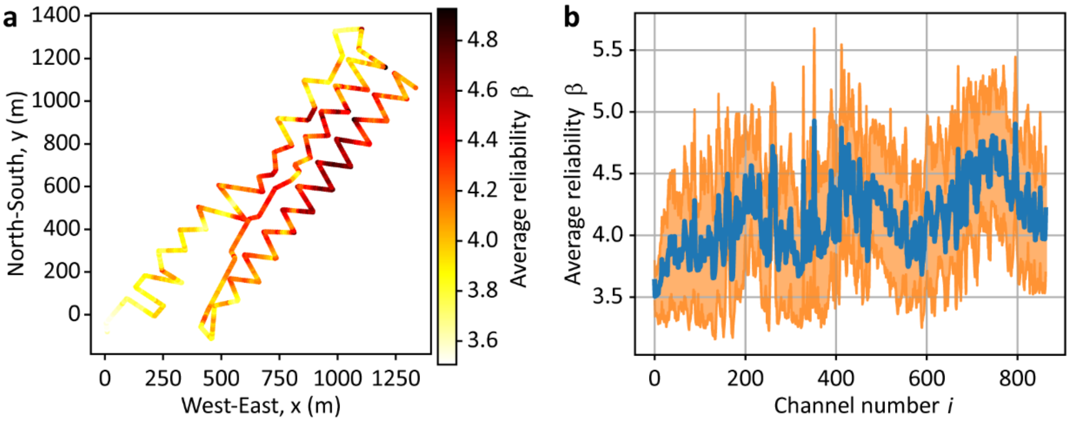

Figure S2. Uneven longitudinal DAS response over the sensing fiber, evaluated over the 55 analyzed source positions. a) Spatial distribution of the average global reliability indicator β. b) Average (blue line) reliability indication β, with an orange area representing the variability range obtained by adding and subtracting one standard deviation from the average value, as a function of the DAS channel number.

Supporting Information 3: Complementary results of beamforming-based acoustic imaging

This section provides additional results to illustrate the clustering effect of selected DAS channels when the acoustic source is near the optical fiber, leading to lower spatial resolution, as shown in Figure 4 of the main text of the manuscript. Figure S3 shows four examples with errors near the median using the 60 most reliable DAS channels. For these cases, when the source is close to the fiber, as shown in Figure S3a and S3b, the most reliable channels also tend to be the closest ones to the source, forming a cluster around it. This causes a loss in resolution as the aperture of the DAS array is not extended along all available channels. In the two examples shown in Figure S3a and S3d, where the sources are far from the fiber, a more scattered selection of channels is yielded, resulting in a better resolution for the image. These trends are similar to the ones observed in Figure 4 of the main text for sources T180 and T141.

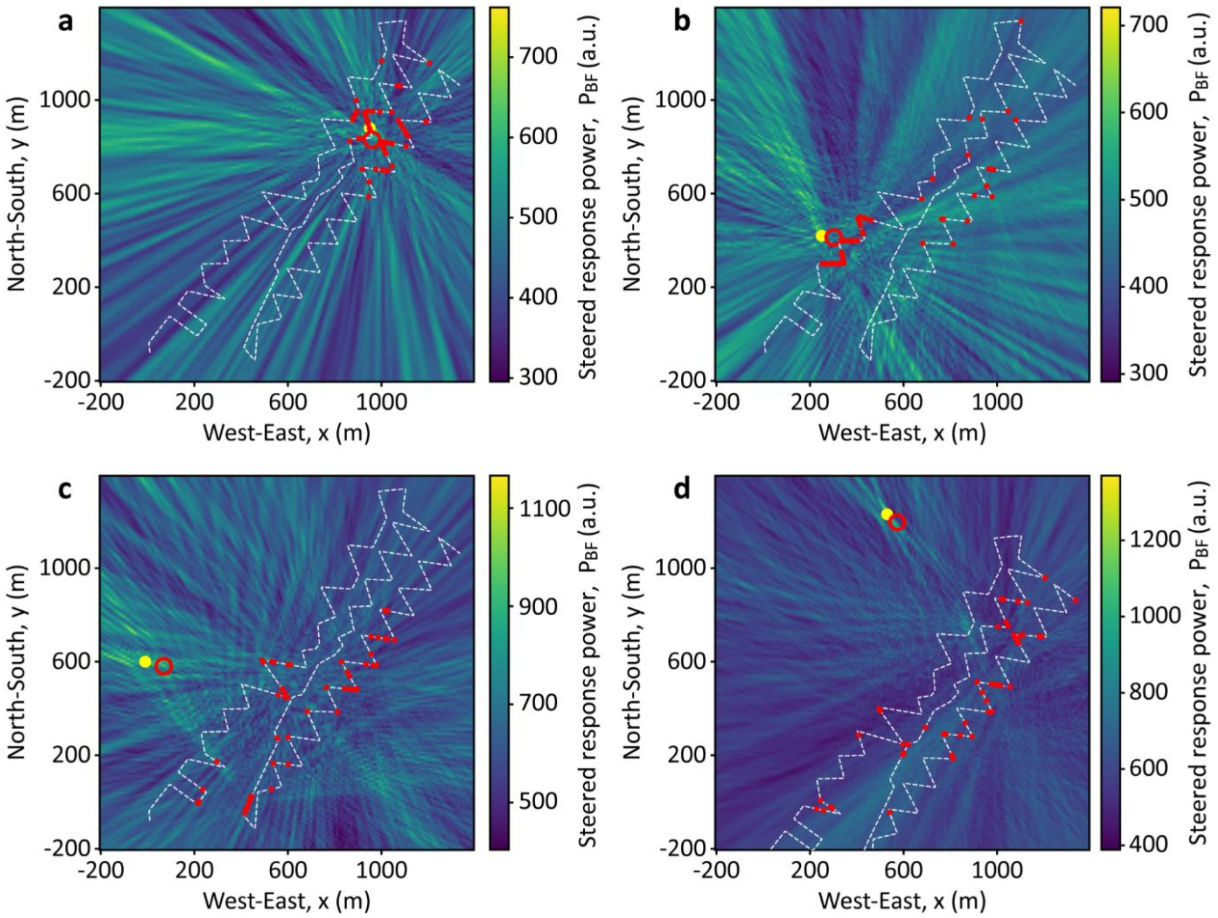

Figure S3. Imaging examples for four very different source locations with estimations errors around the median. Images obtained using the 60 best-quality DAS channels, for a) source T90, with estimated speed: 338 m/s, b) source T127, with estimated speed: 326 m/s, c) source T175, with estimated speed: 328 m/s, and d) source T189, with estimated speed: 326 m/s.

Supporting Information 4: Selection of the optimal number of DAS channels

The process of beam steering in a DAS array to image different regions within the target 2D area results in distinct beamforming responses. Thus, identifying the optimal number of channels is critical to secure accurate acoustic imaging. Selecting channels based on a previously presented approach for single-source localization, which relies on similar reliability indicators, is not suitable in this case. This is because the optimal number of DAS channels for one source location may differ for others, leading to regions with suboptimal steering power responses over the imaged area. The method proposed in this work involves an exhaustive preliminary analysis of the imaged area using different numbers of DAS channels and acoustic source positions distributed throughout space. A statistical analysis is then carried out to

identify the optimal number of channels that minimizes the mean errors of the source location estimations across the entire 2D imaged space.

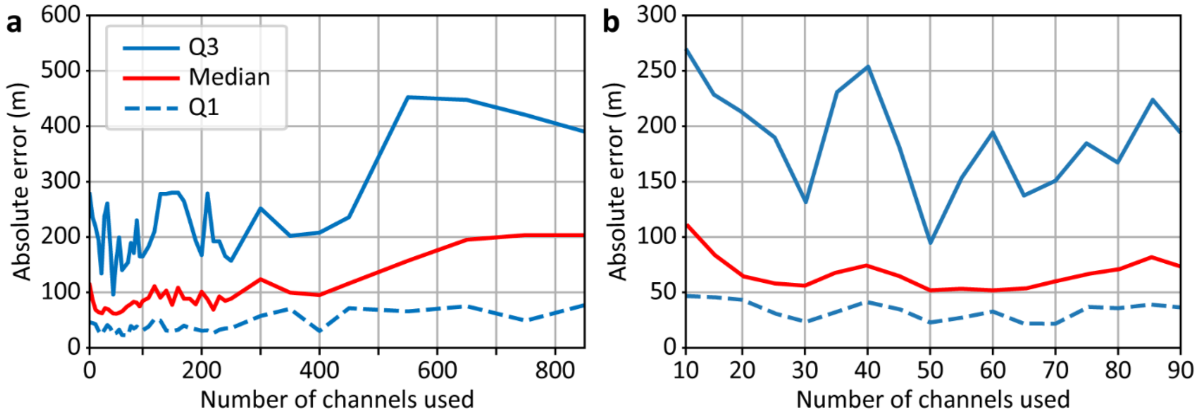

Figure S4. Statistical analysis of the absolute error in source estimation as a function of the number of channels used. Source locations are estimated from acoustic images for the 55 analyzed source positions using different numbers of channels. The median errors (red lines), along with the first quartile (dashed blue line) and third quartile (solid blue line) are shown as a function of the number of channels used. a) Analysis up to 863 channels, and b) analysis focused on the range between 10 and 90 channels, highlighting the range with the lowest errors. Results indicate that the minimum statistical error is achieved with 50 to 60 channels.

Figure S4 presents the statistical analysis of the absolute errors in the estimation of the source location based on acoustic images generated using different numbers of channels, with the 55 source positions depicted in Figure 1. The red lines indicate the median error values, while the dashed and solid blue lines correspond to the first and third quartiles, respectively. Figure S4a shows the errors using up to all 863 DAS channels, indicating that using more than 100 channels tends to increase the errors due to the inclusion of low-quality channels. Figure S4b shows a zoomed-in view, focusing on the error behavior with up to 90 channels, where the third quartile curve indicates that the minimum error occurs when 50 DAS channels are used. However, according to the mean error curve, using between 45 and 75 DAS channels results in nearly the same performance. To secure consistently lower errors for most of the source locations in the imaged area, we opt to use 60 channels, which corresponds to the midpoint of the range showing the lowest median absolute errors. It is important to note that when using a small number of channels, the array does not have sufficient precision to accurately determine the source locations. Increasing the number of channels improves precision but up to an optimal point,

after which including lower-quality DAS channels begins to reduce performance again. Statistically, in the analyzed scenario, the imaging and source localization across the 2D space worsens when more than 70 channels are used, with a significant increase in error statistics beyond 100 channels. However, it is worth noting that adaptive channel selection methods could be explored in future works, as they may offer improved performance in certain scenarios.
